# Neonates with cancer and causes of death; lessons from 615 cases in the SEER databases

**DOI:** 10.1002/cam4.1122

**Published:** 2017-06-22

**Authors:** Ahmad S. Alfaar, Waleed M. Hassan, Mohamed Sabry Bakry, Ibrahim Qaddoumi

**Affiliations:** ^1^ Ophthalmology Department Charité ‐ Universitätsmedizin Berlin (Charité ‐ Berlin Medical University) Berlin Germany; ^2^ Research Department Children's Cancer Hospital Egypt Cairo 57357 Egypt; ^3^ Departments of Oncology St. Jude Children's Research Hospital Memphis Tennessee; ^4^ International Outreach Program St. Jude Children's Research Hospital Memphis Tennessee

**Keywords:** Epidemiology, infancy, neonatal tumors, SEER, undertreatment

## Abstract

Neonatal tumors are rare with no standard treatment approaches to these diseases, and the patients experience poor outcomes. Our aim was to determine the distribution of cancers affecting neonates and compare survival between these cancers and older children.

We analyzed SEER data (1973–2007) from patients who were younger than 2 years at diagnosis of malignancy. Special permission was granted to access the detailed (i.e., age in months) data of those patients. The Chi‐square Log‐rank test was used to compare survival between neonates (aged <1 month) and older children (>1 month to <2 years). We identified 615 neonatal cancers (454 solid tumors, 93 leukemia/lymphoma, and 68 CNS neoplasms). Neuroblastoma was the most common neonatal tumor followed by Germ cell tumors. The 5‐year overall survival (OS) for all neonates was 60.3% (95% CI, 56.2–64.4). Neonates with solid tumors had the highest 5‐year OS (71.2%; 95% CI, 66.9–75.5), followed by those with leukemia (39.1%; 95% CI, 28.3–49.9) or CNS tumors (15%; 95% CI, 5.4–24.6). Except for neuroblastoma, all neonatal tumors showed inferior outcomes compared to that in the older group. The proportion of neonates who died from causes other than cancer was significantly higher than that of the older children (37.9% vs. 16.4%; *P *< 0.0005). In general, the outcome of neonatal cancers has not improved over the last 34 years. The distribution of neonatal cancer is different than other pediatric age groups. Although the progress in neonatal and cancer care over the last 30 years, only death from noncancer causes showed improvement. Studying neonatal tumors as part of national studies is essential to understand their etiology, determine the best treatment approaches, and improve survival and quality of life for those patients.

## Introduction

Neonatal tumors occur during the first month of life and constitute 2% of all childhood cancers 1. Understanding the distribution and behavior of these tumors will enable us to identify the underlying mechanisms, predict survival, and tailor clinical management of each disease. The timing of these neoplasms suggests a genetic origin 2, 3. However, few studies have compared the incidence, survival, or treatment modalities for patients with neonatal tumors 4, 5. The outcomes of neonatal tumors are diverse. Like some leukemias 6, 7, rhabdomyosarcoma 8, and brain tumors 9, 10, some neonatal tumors have poor prognoses; others (neuroblastoma 11 and fibrosarcoma 8) have better ones. Some of the most aggressive tumors (diffuse pontine glioma or high‐grade glioma) spontaneously regress or are cured by surgical resection only 12, 13, supporting Moore's theory that some congenital tumors mature into benign neoplasms 1. Treatments vary according to center and pathology, and many infants with congenital tumors receive no therapy 9. These factors and the rarity of neonatal tumors make it difficult to determine the best treatment and factors influencing survival.

The SEER database provides a unique opportunity to study rare tumors. Thus, we obtained special permission from the SEER administration to analyze monthly data not annual data, as is their standard practice.

## Materials and Methods

We accessed the 17 SEER databases and analyzed data of patients who were <2 years at diagnosis from 1973 to 2007. SEER administrators allowed us to access a custom database (i.e., with age in months) for patients who presented before 2 years of age. Only cases with known age and malignancy are included in the database. Only cases with malignant behavior were included. To extract and analyze data, we used SEER*STAT 8.2.1 and IBM SPSS version 20 software, respectively 14, 15. The SEER registries’ composition and statistical methods are described elsewhere (http://seer.cancer.gov/registries/terms.html). International Classification of Childhood Cancer (ICCC) was used to group the tumors 16. Cancer types were coded using the *International Classification of Diseases for Oncology, 3rd Edition* (Table S1).

For each ICCC group, we calculated frequencies based on sex, race, year of diagnosis, and geographic region. Secondary cancers were extracted if available. To compare survival across eras, we grouped patients into two cohorts: 1973–1990 and 1991–2007. For more detailed analysis, we grouped patients into three cohorts: 1973–1985, 1986–1998, and 1999–2007 to account for changing in treatment eras. Relative frequencies (RFs) were calculated for each ICCC category; within each category, RFs were calculated for gender. Due to the fact that 17 registries did not join SEER at the same decade, these data do not reflect incidence or real change of frequency over time. Five‐year overall survival (OS) was calculated for each ICCC category and subgroup, according sex, race, year of diagnosis, and region. To avoid statistical bias, no statistics were calculated for groups or subgroups with fewer than 10 patients. Although this study has the largest neonatal group of cancer patients, the subgroup analysis of survival should be considered with caution due to the small size of some groups. For this study, *survival plateau* was defined as, “the first point at which the cohort's OS did not change within the subsequent 6 months.” The Chi‐square Log‐rank test was used to compare OS between neonates (<1 month) and older patients (>1 month to <2 years). For simplicity, we grouped intracranial teratomas with the other germ cell tumors in the solid tumors category per ICCC. *P *< 0.05 was considered significant.

## Results

There were 615 (310 males) neonates registered with malignancies. The number of diagnosed patients decreased to 330 at 2 months and 346 at 3 months. The total number of patients registered older than 1 month and <2 years of age were 7804 patients. Solid tumors were the most common diagnosis (*n* = 454), followed by leukemias/lymphomas (*n* = 93), and CNS tumors (*n* = 68). Neuroblastoma was the most prevalent tumor (RF = 0.28), followed by germ cell tumors (RF = 0.27) (Table 1). About 25% of the cases were from the early era (1973–1990), and the rest were from the later era (1991–2007); 502 (81.6%) patients were white.

**Table 1:** Frequency and relative frequency of neonatal cancers according to broad grouping and ICCC categories

ICCC Broad	Grouping	Gender (RF)		Race		Era		Total (RF)
		Male	Female	White	Non‐White	1973–1990	1991–2007	
Leukemias and Lymphomas (*n* = 93)	I Leukemias, myeloproliferative & myelodysplastic diseases:	46 (0.54)^a^	39 (0.46)^a^	69	16	13	72	85 (0.14)^b^
	I(a) Lymphoid leukemias	12	8	15	5	3	17	20 (0.24)^a^
	I(b) Acute myeloid leukemias	19	23	36	6	7	35	42 (0.49)^a^
	I(c) Chronic myeloproliferative diseases	2	0	2	0	1	1	2 (0.02)^a^
	I(d) Myelodysplastic syndrome and other myeloproliferative	1	0	1	0	0	1	1 (0.01)^a^
	I(e) Unspecified and other specified leukemias	12	8	15	5	2	18	20 (0.24)^a^
	II Lymphomas and reticuloendothelial neoplasms	4 (0.50)^a^	4 (0.50)^a^	7	1	1	7	8 (0.01)^b^
	II(a) Hodgkin lymphomas	0	1	1	0	**0**	**1**	1 (0.13)^a^
	II(b) Non‐Hodgkin lymphomas (except Burkitt lymphoma)	1	0	1	0	0	1	1 (0.13)^a^
	II(d) Miscellaneous lymphoreticular neoplasms	3	3	5	1	1	5	6 (0.75)^a^
CNS tumors (*n* = 68)	III CNS and misc intracranial and intraspinal neoplasms	35 (0.51)^a^	33 (0.49)^a^	58	10	22	46	68 (0.11)^b^
	III(a) Ependymomas and choroid plexus tumor	4	2	4	2	2	4	6 (0.09)^a^
	III(b) Astrocytomas	12	15	22	5	11	16	27 (0.40)^a^
	III(c) Intracranial and intraspinal embryonal tumors	13	13	25	1	7	19	26 (0.38)^a^
	III(d) Other gliomas	4	2	5	1	1	5	6 (0.09)^a^
	III(f) Unspecified intracranial and intraspinal neoplasms	2	1	2	1	1	2	3 (0.04)^a^
Other solid tumors (*n* = 451 + 3 patients were not classified by ICCC)	IV Neuroblastoma and other peripheral nervous cell tumors	103 (0.59)^a^	71 (0.41)^a^	143	31	58	116	174 (0.28)^b^
	IV(a) Neuroblastoma and ganglioneuroblastoma	103	70	142	31	58	115	173 (0.99)^a^
	IV(b) Other peripheral nervous cell tumors	0	1	1	0	0	1	1 (0.01)^a^
	V Retinoblastoma	13 (0.48)^a^	14 (0.52)^a^	21	6	7	20	27 (0.04)^b^
	VI(a) Nephroblastoma and other nonepithelial renal tumors	9 (0.56)^a^	7 (0.44)^a^	14	2	10	6	16 (0.03)^b^
	VII(a) Hepatoblastoma	4 (0.57)^a^	3 (0.43)^a^	6	1	3	4	7 (0.01)^b^
	IX Soft tissue and other extraosseous sarcomas	27 (0.53)^a^	24 (0.47)^a^	43	8	16	35	51 (0.08)^b^
	IX(a) Rhabdomyosarcomas	11	8	17	2	8	11	19 (0.37)^a^
	IX(b) Fibrosarcomas, peripheral nerve & other fibrous	9	13	17	5	7	15	22 (0.43)^a^
	IX(d) Other specified soft tissue sarcomas	7	3	9	1	1	9	10 (0.20)^a^
	X Germ cell & trophoblastic tumors & neoplasms of gonads	63 (0.38)^a^	105 (0.63)^a^	134	34	23	143	168 (0.27)^b^
	X(a) Intracranial & intraspinal germ cell tumors	7	13	17	3	0	20	20 (0.12)^a^
	X(b) Extracranial & extragonadal germ cell tumors	55	91	115	31	23	123	146 (0.87)^a^
	X(c) Malignant gonadal germ cell tumors	1	0	1	0	0	1	1 (0.01)^a^
	X(e) Other and unspecified malignant gonadal tumors	0	1	1	0	0	1	1 (0.01)^a^
	XI Other malignant epithelial neoplasms and melanomas	1 (0.25)^a^	3 (0.75)^a^	2	2	2	2	4 (0.01)^b^
	XI(d) Malignant melanomas	1	2	2	1	2	1	3 (0.75)^a^
	XI(f) Other and unspecified carcinomas	0	1	0	1	0	1	1 (0.25)^a^
	XII Other and unspecified malignant neoplasms	2 (0.50)^a^	2 (0.50)^a^	4	0	2	2	4 (0.01)^b^
	XII(a) Other specified malignant tumors	1	1	2	0	0	2	2 (0.50)^a^
	XII(b) Other unspecified malignant tumors	1	1	2	0	2	0	2 (0.50)^a^
	Not classified by ICCC	3	0	1	2	1	2	3
	Grand Total	310 (0.50)^b^	305 (0.50)	502	113	158	457	

RF, relative frequency; RTh, radiation therapy; Surg, surgery.

a Relative frequency was calculated as a fraction of the ICCC group.

b Relative frequency was calculated as a fraction of total cases.

### General outcome

**Figure 1:**
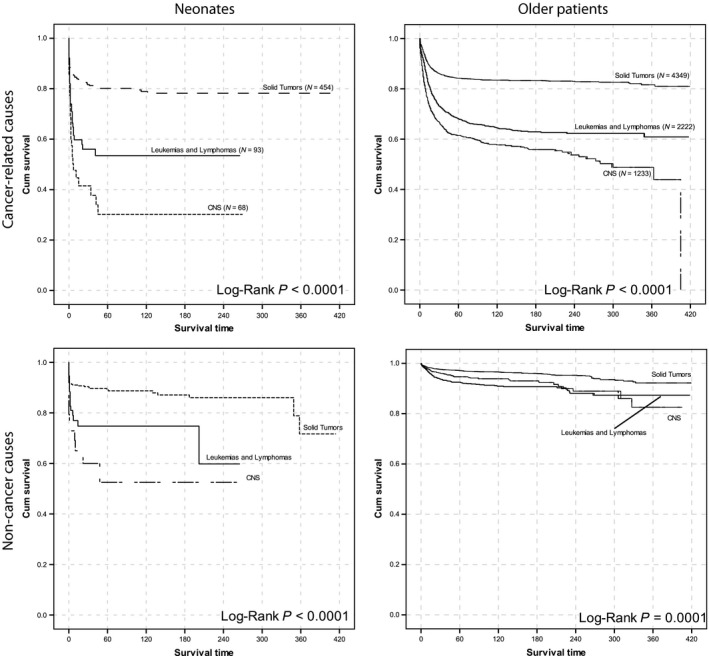
Survival of neonatal patients and older patients grouped based on ICCC categories. Cancer‐specific and noncancer‐related deaths are separated.

The 5‐year OS for all neonates was 60.3% (95% CI, 56.2–64.4). Patients with solid tumors had the highest 5‐year OS (71.2%; 95% CI, 66.9–75.5), followed by leukemia (39.1%; 95% CI 28.3–49.9), and CNS tumors (15%; 95% CI, 5.4–24.6) (Fig. 1, Table 2, Figure S1 and S2). Except for neuroblastoma, all of the neonatal tumors showed significantly inferior outcomes compared to that in older patients. Lymphoma and hepatoblastoma data were removed from further analysis because those subgroups included fewer than 10 cases.

**Figure 2:**
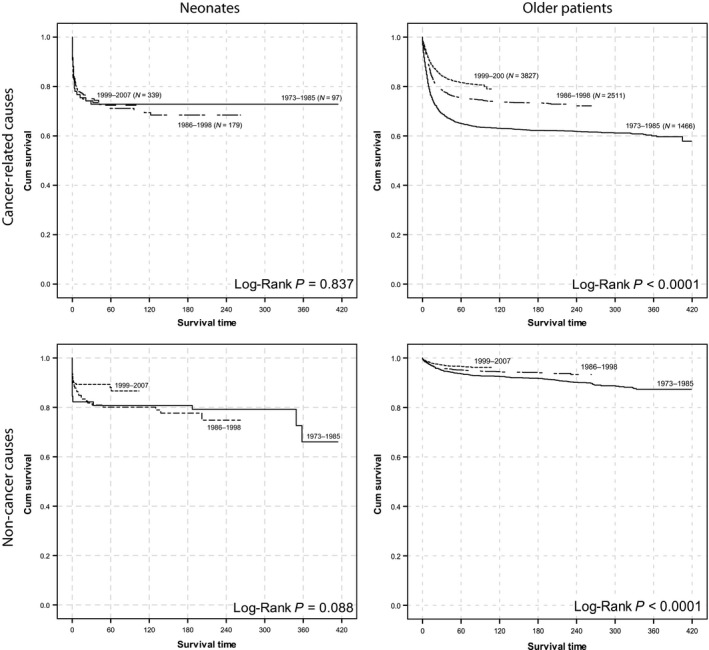
Survival of neonatal patients and older patients during the last three decades. Cancer‐specific and noncancer‐related deaths are separated.

**Figure 3:**
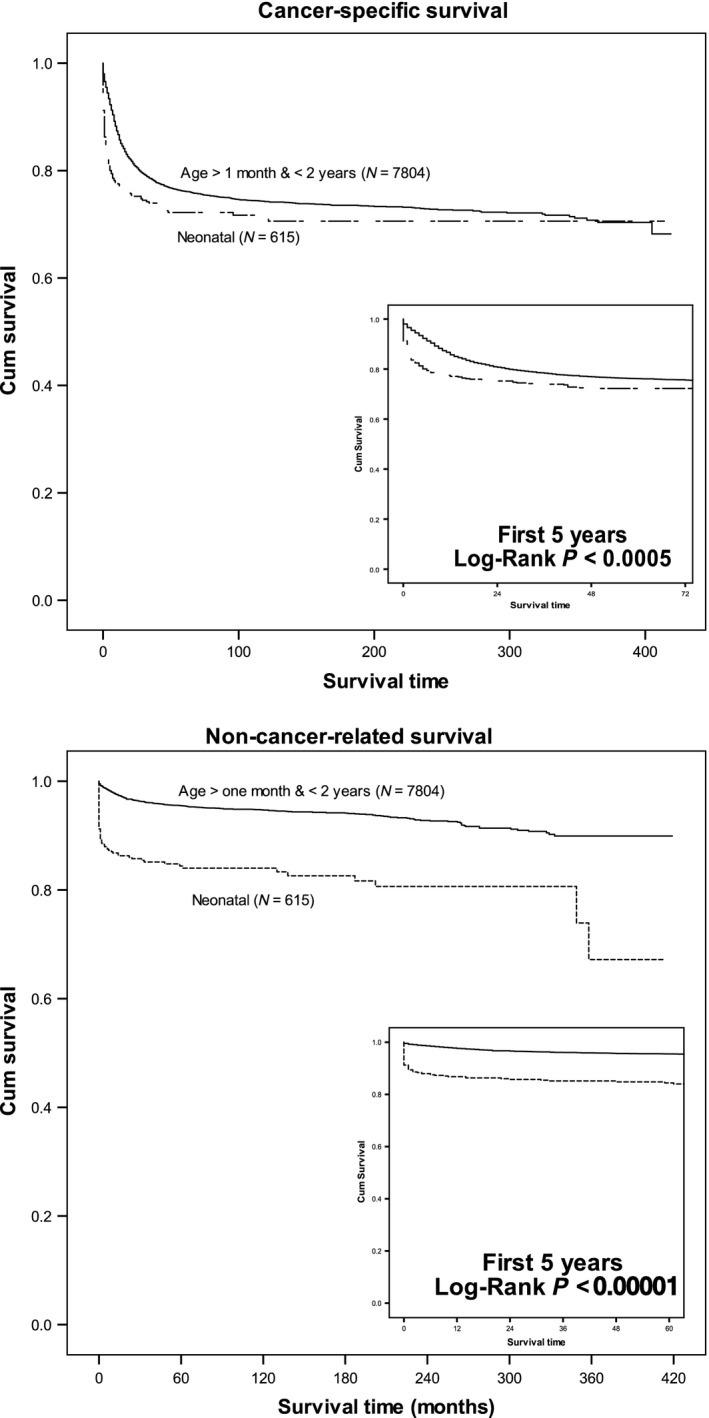
Comparison of cancer‐specific and noncancer‐related survival between neonates and older patients with cancer.

The proportion of neonates who died of noncancer causes was higher than that of older patients (37.9% vs. 16.4%; Chi–square *P* < 0.0005) (Tables S2–S4). Cancer–specific and noncancer‐related OS was significantly better in older patients from the later era than in those from the earlier era; however, OS did not improve in neonates (Fig. 2). Most neonates died within 1 month of diagnosis, either from cancer (36.2%) or other causes (59.3%) (data not shown, Fig. 3, Table 3). Interventions across eras are presented in Figure S3.

**Table 2:** Five‐year overall survival of patients in each ICCC category with common subtypes having cases more than the predetermined cutoff and according to sex, race, era of diagnosis, geographic region and treatment received

Broad Grouping	ICCC	Gender (95% CI)		Race (95% CI)		Era of diagnosis (95% CI)		Total (95% CI)	>1 month–2 years
		Male	Female	White	Non ‐ White	1973–1990	1991–2007		
Leukemias	I Leukemias, myeloproliferative & myelodysplastic diseases:	41.6 (25.9–57.3)	37.7 (22.2–53.2)	39.9 (27.4–52.4)	36.5 (12.4–60.6)	43.3 (15.3–71.3)	38.4 (26.2–50.6)	39.1 (27.9–50.3)	62.8 (60.4–65.2)^a^
	I(a) Lymphoid leukemias							25 (2.9–47.1)	
	I(b) Acute myeloid leukemias							48.6 (33.1–64.1)	
	I(e) Unspecified and other specified leukemias							36.8 (13.7–59.9)	
CNS tumors	III CNS and misc intracranial and intraspinal neoplasms	17 (2.9–31.1)	12.4 (0–25.1)	19.3 (8.1–30.5)		9.1 (0–21.1)	19.1 (5.6–32.6)	15 (5.4–24.6)	58.5 (55.6–61.4)^a^
	III(b) Astrocytomas							22.9 (5.7–40.1)	
	III(c) Intracranial and intraspinal embryonal tumors							9.4 (0–21.6)	
Other solid tumors	IV Neuroblastoma and other peripheral nervous cell tumors	79.7 (71.7–87.7)	72.5 (61.9–83.1)	77.8 (70.9–84.7)	70.1 (52.7–87.5)	70.6 (58.8–82.4)	80.1 (72.7–87.5)	76.8 (70.3–83.3)	78.6 (76.4–80.8)^a^
	V Retinoblastoma	83.9 (63.5–100)	100	–	–	–	–	91.7 (80.5–100)	95.7 (94.1–97.3)^b^
	VI(a) Nephroblastoma and other nonepithelial renal tumors							62.5 (38.8–86.2)	87.9 (85.4–90.4)^a^
	IX Soft tissue and other extraosseous sarcomas	46.7 (25.7–67.7)	59.8 (39.2–80.4)	–	–	62.5 (38.8–86.2)	45 (25.6–64.4)	52.4 (37.5–67.3)	71.5 (67–76)^a^
	IX(a) Rhabdomyosarcomas							36.3 (12.8–59.8)	
	IX(b) Fibrosarcomas, peripheral nerve & other fibrous							76 (57.4–94.6)	
	IX(d) Other specified soft tissue sarcomas							31.1 (0–64)	
	X Germ cell & trophoblastic tumors & neoplasms of gonads	61.9 (48–75.8)	71.7 (62.7–80.7)	68.9 (60.5–77.3)	66.9 (49.7–84.1)	51.2 (30.4–72)	71.3 (63.3–79.3)	68.3 (60.7–75.9)	84 (79.9–88.1)^a^
	X(a) Intracranial & intraspinal germ cell tumors							25 (6–44)	
	X(b) Extracranial & extragonadal germ cell tumors	63.1 (47.6–78.6)	80.7 (72.1–89.3)	75.1 (66.5–83.7)	73.5 (56.3–90.7)	51.2 (30.4–72)	79.2 (71–87.4)	74.5 (66.7–82.3)	
	Grand Total	59.3 (53.4–65.2)	61.3 (55.6–67)	60.8 (56.3–65.3)	57.2 (47.2–67.2)	57.3 (49.5–65.1)	61.3 (56.4–66.2)	60.3 (56.2–64.4)	72.7 (71.7–73.7)^a^

RTh, radiation therapy; Surg, surgery.

*P* ≤ 0.001.

a No statistics were calculated because the group had fewer than 10 patients.

b *P* = 0.043.

**Table 3:** Proportion of patients who died of either noncancer‐ or cancer‐related causes

Age Group	Cause of death^a^		Total
	Noncancer *n* (%)	Cancer *n* (5)	
Neonate	91 (37.9)	149 (62.1)	240
Older	349 (16.4)	1780 (83.6)	2129
Total	440 (18.6)	1929 (81.4)	2369

a Pearson Chi‐Square = 66, *P* < 0.0005.

### Solid tumors

#### Neuroblastoma

Neuroblastoma was the most common neonatal tumor (*n* = 174, RF = 0.28) in the SEER database. Male neonates comprised 60% of patients. The 5–year OS was 76.8% (95% CI, 70.3–83.3), with a survival plateau after 30 months (Table S6). There was no difference in OS based on sex, race, era, or region (Table 2). In cases of neuroblastoma, there was no significant difference in survival between neonates and the older group (Log–rank *P* = 0.062) (Table 2).

The adrenal gland was the most common primary tumor site, occurring in almost 50% of neuroblastoma cases, followed by connective tissue, the retroperitoneum, and the mediastinum. Adrenal gland tumors appeared to have the lowest 5–year OS (69.7%; 95% CI, 59.7–79.7), but the difference was not significant (Table S5). No secondary cancers occurred in neonates with neuroblastoma.

#### Retinoblastoma

The SEER records revealed 27 cases of retinoblastoma (RF = 0.04). The 5–year OS of neonates with retinoblastoma was 91.7% (95% CI, 80.5–100) (Table 2), with a survival plateau after 6 months (Table S6). This group experienced the highest OS among the neonatal disease subgroups. The OS of older patients who had retinoblastoma was significantly better than that of the neonates (Log‐rank *P* = 0.043). Only one patient with retinoblastoma had secondary cancer.

#### Nephroblastoma

Renal tumors were also rare (*n* = 16; RF = 0.03). The 5–year OS for neonates with nephroblastoma was 62.5% (95% CI, 38.8–86.2) (Table 2), with a survival plateau after 6 months (Table S6). Older patients had a significantly higher OS (87.9%; 95% CI, 85.4–90.4; *P* < 0.001).

#### Soft‐tissue tumors

Fifty‐one neonates had soft‐tissue tumors (RF = 0.08)(Table 1). The 5‐year OS for these patients was 52.4% (95% CI, 37.5–67.3), with a survival plateau after 18 months (Tables 2, S6). There was no difference in OS on the basis of sex, race, era, or region. Older patients with soft‐tissue tumors had a significantly higher OS (71.5%; 95% CI, 67–76; *P* = 0.001).

Rhabdomyosarcomas and fibrosarcomas comprised the main histologies of soft‐tissue tumors in this study. Patients with fibrosarcomas had a 5‐year OS of 76% (95% CI, 57.4–94.6), which was significantly higher than that of patients with rhabdomyosarcomas (36.3%; 95% CI, 12.8–59.8) (Table 2). Connective tissue was the most common primary site of soft‐tissue tumors. Fibrosarcomas occurred mainly in the limbs, and rhabdomyosarcomas occurred mainly in the head and neck (data not shown).

#### Germ cell tumors

Germ cell tumors were the second‐most common neonatal tumors in the SEER database (RF = 0.27); 168 neonates had germ cell tumors, 20 of which were CNS tumors. Of the remaining 148 cases, two were gonadal and 146 were extragonadal. Female neonates comprised 62% of cases (Table 1). The 5‐year OS of neonates with germ cell tumors was 68.3% (95% CI, 60.7–75.9), with a survival plateau after 12 months (Tables 2 and S6). The 5‐year OS for neonates with extracranial or extragonadal germ cell tumors was 74.5% (95% CI, 66.7–82.3), which was significantly higher than that for neonates with CNS tumors (25%; 95% CI, 6–44) (Table 2). The 5‐year OS did not differ based on sex, race, era, or region. Older patients had a significantly higher OS than did the neonates (84%; 95% CI, 79.9–88.1; *P* < 0.001). Two patients with germ cell tumors had secondary cancers.

### Leukemias

Leukemias comprised 14% of our cohort, of which 49% were acute myeloid leukemia (AML), and 24% were acute lymphocytic leukemia (ALL) (Table 1). The 5‐year OS for all leukemia cases was 39.1% (95% CI, 27.9–50.3), with a survival plateau after 24 months (Table 2 and S6). Patients with AML appeared to have a higher 5‐year OS than did those with ALL, but the difference was not significant (*P* = 0.403). Survival of the older patients with leukemia was 62.8% (95% CI, 60.4–65.2), which was significantly higher (*P* < 0.001) than that of the neonates (Table 2). There was no difference in 5‐year OS among neonates based on sex, race, era, or region. One patient suffered a secondary cancer.

### CNS tumors

CNS tumors were the fourth‐most common tumors affecting neonates in this study (RF = 0.11; Table 1, Table S7). Astrocytoma was the most common histology (40%), followed by intracranial and intraspinal embryonal tumors (38%). Of the 26 intracranial embryonal tumors, 17 were PNET, and seven were medulloblastomas (Table S1). The 5‐year OS was 15% (95% CI, 5.4–24.6), with a survival plateau after 24 months (Tables 2, S6). Patients with astrocytomas had a 5‐year OS of 22.9% (95% CI, 5.7–40.1), which appeared to be higher than that of those with intracranial or intraspinal embryonal tumors (9.4%; 95% CI, 0–21.6) (Table 1), but the difference was not significant. Survival of the older patients was 58.5% (95% CI, 55.6–61.4), which was significantly higher than that of the neonates (*P* < 0.001) (Table 2).

## Discussion

Tumors rarely arise during the first month of life. In the U.K., only 303 neonates with cancer were reported over a 3–decade period 4. To the best of our knowledge, this study represents the largest cohort of neonatal cancers ever studied. Nevertheless, the increasing number of patients over time is related to the fact that the 17 registries are enrolled in the SEER program over the period between 1973 and 2000 and pooled their patients gradually to the database 17.

Neonates with tumors experienced significantly inferior outcome compared with that of older patients who had the same disease, except for those with neuroblastoma. We believe the one cause of this difference could be treatment denial for newborns. This hypothesis is supported by the higher rate of death due to causes other than cancer among neonates in our study (Table S4). In our study 50.1% of children died of causes other than cancer (27% from congenital anomalies and 23.1% from perinatal conditions as defined in ICD‐10‐CM Guidelines 18). This highlights another limitation of SEER data and our study and makes concrete conclusions difficult but it also underscores again our plea for further support and wider access to detailed data from patients reported on SEER.

Many neonates with tumors probably die of cardiac and/or respiratory dysfunction secondary to their untreated cancer. One possible explanation is that these infants are treated in neonatal intensive care units, and the decision to not treat their disease is occasionally made without consulting a pediatric oncologist. This explanation requires further investigation and conducting wider retrospective and prospective studies. Unfortunately, the SEER does not provide public access to information (to the time of writing this article) on chemotherapy regimens, so we cannot conclude anything about the prevalence of that approach to treating neonates.

Other studies have documented withholding therapy from neonates with cancer. In Isaacs’ study 9 of 154 children with CNS tumors, 120 were not offered any treatment, but the 34 who received any kind of therapy experienced superior outcome. However, no quality‐of‐life data were provided.

We are not proposing aggressive treatment of all neonates, especially those with CNS tumors, without consideration of quality of life, or in patients with serious comorbidities or congenital anomalies (Table S4) as this may cause more harm than benefit. In a study from Japan, 76% of neonates who survived CNS tumors suffered mental retardation 10. We propose that special attention be given to neonates to determine the optimal therapy with minimal toxicity (e.g., differentiating agents) that can be administered 1, 12, 13.

As reported by others 4, we found a disturbing lack of improvement in outcome over the last 34 years in all of the neonatal tumors we investigated. The fact that even with the major advances in medicine, oncologic treatment, supportive care, and neonatology care the outcome in the third period is not much different than 30 years earlier underscores that the major hindrance is related to age group and not other external factors. In older children, survival of every tumor type improved every decade. We believe the lack of outcome improvement, despite all the medical advancements, could result from depriving many neonates effective (or any) therapy. For each tumor group, the outcome of neonates was worse than that of older children. A similar observation was made in the U.K. neonatal cohort, even in cases in which the tumors were associated with a good prognosis 4.

In our study, neuroblastoma was the most common neonatal tumor. Most other neonatal cancer studies have reported teratomas as the most common neoplasm, followed by neuroblastoma 1, 5. This difference may reflect the fact that the SEER registry does not report mature teratomas. The 5‐year OS in our study was 76.8%, which is comparable to that in other studies (74% 19 and 88.3% 11). In addition, Isaacs reported improved survival between cases before 1983 and those diagnosed thereafter. Era did not affect survival in our study.

The second‐most common tumor in our study was germ cell tumors. The SEER database does not include mature or benign tumors before 2004; therefore, it is difficult to compare the outcome of those diseases with that of such tumors in other studies. Acute leukemias are rare in neonates, compared to their incidence in older children 20. The distinction between congenital and neonatal leukemia is arbitrary, and most reports discuss congenital, neonatal, and infantile leukemias as a single group 6, 21. Our study confirmed that patients with neonatal leukemias have a much poorer prognosis than older patients and less improvement in outcome, especially for those with ALL 4, 6, 7. AML was the more common diagnosis than ALL in neonates, which was the case in earlier reports 21, 22. The prognosis of neonates with AML was also better than that of neonates with ALL, which supports results from all other reports 7, 21 and contradicts what is seen in older children. This difference might reflect the fact that neonatal AML is closely related to transient myeloproliferative disorder, which shows spontaneous remission in most of cases.

Recently, an update for WHO Classification for Pediatric Brain Tumors has removed PNET as an entity from the classification and it was integrated in embryonal tumors. The diagnoses of this group became dependent on the presence C19MC amplification 23. The new classification cannot be retrospectively implemented on the current SEER data without access to their histopathology and immunohistochemistry panels.

Neonates with cancer died faster than older children with cancer either from cancer or noncancer‐related causes. However, noncancer‐related deaths differentiated survival between neonatal and older patients. The 30‐year OS of patients who experienced neonatal cancers equaled that of those who experienced cancer at an older age. Survival plateaus can help researchers interpret the crucial periods for each tumor entity. We believe that some cancer‐related deaths that occurred during the first month of life can be attributed to insufficient supportive care. Askin 24 outlined a supportive care plan for patients with neonatal cancer that we believe should be further investigated.

This research could not have been done without the SEER administrative team's approval. We believe that the SEER data is a crucial tool toward increasing our understanding of rare cancers, although its limitations 25. For example, it would be interesting to investigate the incidence of second cancers and familial cancers in these children and their families because many believe neonatal tumors are indicative of genetic predisposition 2. Previous studies have shown a correlation between congenital anomalies and childhood cancers 26, 27, 28. We believe and propose that improving funding for SEER to acquire data on comorbidities, tumor biology, details of received treatments and even contacting surviving patients and their families for genetic testing or long term effects studies will open the flood gates of unlimited research opportunities. The concept of wider access to data will only enhance science 29, 30.

## Conflict of Interest

No conflict of interest to be disclosed by authors.

## Supporting information


**Table S1.** ICCC categories and ICD‐O‐3 codes used in the study and frequency of patients (*n*).
**Table S2.** Comparison of the causes of death across the age groups.
**Table S3.** Comparison of the probabilities of survival of cancer or noncancer causes across the age groups.
**Table S4.** Comparisons of the causes of death (other than the primary cancer) between patients who were neonates at diagnosis and those who were older (>1 month and <2 years).
**Table S5.** The relative frequency of neuroblastoma diagnoses and 5‐year overall survival of patients with that disease.
**Table S6.** Survival plateaus* for each ICCC category in the neonatal group.
**Table S7.** Common sites of primary disease identified by the WHO 2008.
**Figure S1.** Survival by study groups by year of diagnosis. An “event” is defined as death due to cancer OR other causes.
**Figure S2.** Comparison of cancer‐ vs. general survival on the basis of age at diagnosis and tumor category.
**Figure S3.** Comparisons of the pattern of intervention between neonatal and older patients over the last 3 decades.
