# Supplementary material for: Neonates with cancer and causes of death; lessons from 615 cases in the SEER databases
Source: Cancer Med. 2017 Jun 22;6(7):1817–26. doi: 10.1002/cam4.1122 (PMC5504346; doi:10.1002/cam4.1122)
Supplement: Supplementary file 1 — Table S1. ICCC categories and ICD‐O‐3 codes used in the study and frequency of patients (n). Table S2. Comparison of the causes of death across the age groups. Table S3. Comparison of the probabilities of survival of cancer or noncancer causes across the age groups. Table S4. Comparisons of the causes of death (other than the primary cancer) between patients who were neonates at diagnosis and those who were older (>1 month and <2 years). Table S5. The relative frequency of neuroblastoma diagnoses and 5‐year overall survival of patients with that disease. Table S6. Survival plateaus* for each ICCC category in the neonatal group. Table S7. Common sites of primary disease identified by the WHO 2008. Figure S1. Survival by study groups by year of diagnosis. An “event” is defined as death due to cancer OR other causes. Figure S2. Comparison of cancer‐ vs. general survival on the basis of age at diagnosis and tumor category. Figure S3. Comparisons of the pattern of intervention between neonatal and older patients over the last 3 decades. [file CAM4-6-1817-s001.docx]

Supplementary Data

# Table of Contents

[Table of Contents 1](#_Toc479025012)

[Supplementary Table 1. 2](#_Toc479025013)

[Supplementary Table 2. 5](#_Toc479025014)

[Supplementary Table 3. 6](#_Toc479025015)

[Supplementary Table 4. 7](#_Toc479025016)

[Supplementary Table 5. 9](#_Toc479025017)

[Supplementary Table 6. 10](#_Toc479025018)

[Supplementary Table 7. 11](#_Toc479025019)

[Supplementary Figure 1. 12](#_Toc479025020)

[Supplementary Figure 2. 13](#_Toc479025021)

[Supplementary Figure 3. 14](#_Toc479025022)

# Supplementary Table 1.

ICCC categories and ICD-O-3 codes used in the study and frequency of patients (n).

| **Leukemias and Lymphomas** | **n** | **CNS Tumors** | **n** | **Other Solid Tumors** | **n** |
| --- | --- | --- | --- | --- | --- |
|  |  |  |  |  |  |
| **I(a) Lymphoid leukemias** | **20** | **III(a) Ependymomas and choroid plexus tumor** | **6** | **IV(a) Neuroblastoma and ganglioneuroblastoma** | **173** |
| 9835/3: Precursor cell lymphoblastic leukemia, NOS | 16 | 9390/3: Choroid plexus papilloma, malignant | 2 | 9490/3: Ganglioneuroblastoma | 3 |
| 9836/3: Precursor B-cell lymphoblastic leukemia | 4 | 9391/3: Ependymoma, NOS | 2 | 9500/3: Neuroblastoma, NOS | 170 |
| **I(b) Acute myeloid leukemias** | **42** | 9392/3: Ependymoma, anaplastic | 2 | **IV(b) Other peripheral nervous cell tumors** | **1** |
| 9861/3: Acute myeloid leukemia | 21 | **III(b) Astrocytomas** | **27** | 9501/3: Medulloepithelioma, NOS | 1 |
| 9867/3: Acute myelomonocytic leukemia | 3 | 9380/3: Glioma, malignant | 1 | **V Retinoblastoma** | **27** |
| 9872/3: Acute myeloid leukemia, minimal differentiation | 2 | 9400/3: Astrocytoma, NOS | 11 | 9510/3: Retinoblastoma, NOS | 26 |
| 9874/3: Acute myeloid leukemia with maturation | 1 | 9401/3: Astrocytoma, anaplastic | 3 | 9511/3: Retinoblastoma, differentiated | 1 |
| 9891/3: Acute monocytic leukemia | 7 | 9420/3: Fibrillary astrocytoma | 1 | **VI(a) Nephroblastoma and other nonepithelial renal tumors** | **16** |
| 9910/3: Acute megakaryoblastic leukemia | 8 | 9421/3: Pilocytic astrocytoma, malignant | 2 | 8960/3: Nephroblastoma, NOS | 13 |
| **I(c) Chronic myeloproliferative diseases** | **2** | 9440/3: Glioblastoma, NOS | 8 | 8963/3: Malignant rhabdoid tumor | 3 |
| 9863/3: Chronic myeloid leukemia, NOS | 2 | 9442/3: Gliosarcoma | 1 | **VII(a) Hepatoblastoma** | **7** |
| **I(d) Myelodysplastic syndrome and other myeloproliferative** | **1** | **III(c) Intracranial and intraspinal embryonal tumors** | **26** | 8970/3: Hepatoblastoma | 7 |
| 9946/3: Juvenile myelomonocytic leukemia | 1 | 9470/3: Medulloblastoma, NOS | 7 | **IX(a) Rhabdomyosarcomas** | **19** |
| **I(e) Unspecified and other specified leukemias** | **20** | 9473/3: Primitive neuroectodermal tumor | 17 | 8900/3: Rhabdomyosarcoma, NOS | 2 |
| 9800/3: Leukemia, NOS | 8 | 9503/3: Neuroepithelioma, NOS | 1 | 8910/3: Embryonal rhabdomyosarcoma | 13 |
| 9801/3: Acute leukemia, NOS | 9 | 9508/3: Atypical teratoid/rhabdoid tumor | 1 | 8920/3: Alveolar rhabdomyosarcoma | 4 |
| 9805/3: Acute biphenotypic leukemia | 2 | **III(d) Other gliomas** | **6** | **IX(b) Fibrosarcomas, peripheral nerve & other fibrous** | **22** |
| 9930/3: Myeloid sarcoma | 1 | 9380/3: Glioma, malignant | 4 | 8810/3: Fibrosarcoma, NOS | 4 |
| **II(a) Hodgkin lymphomas** | **1** | 9382/3: Mixed glioma | 1 | 8813/3: Fascial fibrosarcoma | 1 |
| 9663/3: Hodgkin lymphoma, nodular sclerosis, NOS | 1 | 9430/3: Astroblastoma | 1 | 8814/3: Infantile fibrosarcoma | 11 |
| **II(b) Non-Hodgkin lymphomas (except Burkitt lymphoma)** | **1** | **III(f) Unspecified intracranial and intraspinal neoplasms** | **3** | 9150/3: Hemangiopericytoma, malignant | 4 |
| 9729/3: Precursor T-cell lymphoblastic lymphoma | 1 | 8000/3: Neoplasm, malignant | 3 | 9540/3: Malignant peripheral nerve sheath tumor | 1 |
| **II(d) Miscellaneous lymphoreticular neoplasms** | **6** |  |  | 9580/3: Granular cell tumor, malignant | 1 |
| 9740/3: Mast cell sarcoma | 1 |  |  | **IX(d) Other specified soft tissue sarcomas** | **10** |
| 9741/3: Malignant mastocytosis | 1 |  |  | 8832/3: Dermatofibrosarcoma, NOS | 2 |
| 9754/3: Langerhans cell histiocytosis, disseminated | 4 |  |  | 8890/3: Leiomyosarcoma, NOS | 1 |
|  |  |  |  | 8963/3: Malignant rhabdoid tumor | 4 |
|  |  |  |  | 9130/3: Hemangioendothelioma, malignant | 2 |
|  |  |  |  | 9231/3: Myxoid chondrosarcoma | 1 |
|  |  |  |  | **X(a) Intracranial & intraspinal germ cell tumors** | **20** |
|  |  |  |  | 9080/3: Teratoma, malignant, NOS | 20 |
|  |  |  |  | **X(b) Extracranial & extragonadal germ cell tumors** | **146** |
|  |  |  |  | 9070/3: Embryonal carcinoma, NOS | 2 |
|  |  |  |  | 9071/3: Yolk sac tumor | 4 |
|  |  |  |  | 9080/3: Teratoma, malignant, NOS | 134 |
|  |  |  |  | 9081/3: Teratocarcinoma | 3 |
|  |  |  |  | 9085/3: Mixed germ cell tumor | 3 |
|  |  |  |  | **X(c) Malignant gonadal germ cell tumors** | **1** |
|  |  |  |  | 9071/3: Yolk sac tumor | 1 |
|  |  |  |  | **X(e) Other and unspecified malignant gonadal tumors** | **1** |
|  |  |  |  | 8631/3: Sertoli-Leydig cell tumor, poorly differentiated | 1 |
|  |  |  |  | **XI(d) Malignant melanomas** | **3** |
|  |  |  |  | 8720/3: Malignant melanoma, NOS | 3 |
|  |  |  |  | **XI(f) Other and unspecified carcinomas** | **1** |
|  |  |  |  | 8071/3: Squamous cell carcinoma, keratinizing, NOS | 1 |
|  |  |  |  | **XII(a) Other specified malignant tumors** | **2** |
|  |  |  |  | 8936/3: Gastrointestinal stromal sarcoma | 1 |
|  |  |  |  | 8971/3: Pancreatoblastoma | 1 |
|  |  |  |  | **XII(b) Other unspecified malignant tumors** | **2** |
|  |  |  |  | 8000/3: Neoplasm, malignant | 2 |
|  |  |  |  | **Not classified by ICCC or in situ** | **3** |
|  |  |  |  | 8814/3: Infantile fibrosarcoma | 1 |
|  |  |  |  | 8963/3: Malignant rhabdoid tumor | 1 |
|  |  |  |  | 9364/3: Peripheral neuroectodermal tumor | 1 |

**Abbreviations:** ICCC, International Classification of Childhood Cancer; *ICD-O-3, International Classification of Diseases for Oncology, 3^rd^ edition*; NOS, not otherwise specified

Bold typeface indicates a category.

# Supplementary Table 2.

Comparison of the causes of death across the age groups.

| **Age at Diagnosis (mos)** | | **No. of patients** | **Cause of death** | | **No. of living patients n (%)^*^** |
| --- | --- | --- | --- | --- | --- |
|  |  |  | **Noncancer-related n (%)** | **Cancer-related**  **n (%)** |  |
| <1 | 615 | | 91 (14.8) | 149 (24.2) | 375 (61) |
| >1-2 | 330 | | 20 (6.1) | 75 (22.7) | 235 (71.2) |
| >2-3 | 346 | | 24 (6.9) | 77 (22.3) | 245 (70.8) |
| >3-<24 | 7128 | | 305 (4.3) | 162 (22.8) | 5195 (72.9) |
| Total | 8419 | | 440 (5.2) | 1929 (22.9) | 6050 (71.9) |

^*^The data reflect the number of living patients as of 8 August 2014.

# Supplementary Table 3.

Comparison of the probabilities of survival of cancer or noncancer causes across the age groups

| Cause of death | Age  Group | Probability of survival at different times after diagnosis | | | | | |
| --- | --- | --- | --- | --- | --- | --- | --- |
|  |  | 1 mos | 2 mos | 3 mos | 4 mos | 1 y | 5 y |
| Cancer | Neonates | .912 | .863 | .836 | .824 | .775 | .722 |
| Cancer | Older patients | .980 | .966 | .955 | .945 | .867 | .762 |
| Noncancer | Neonates | .912 | .894 | .886 | .884 | .868 | .844 |
| Noncancer | Older patients | .995 | .992 | .991 | .988 | .978 | .955 |

# Supplementary Table 4.

Comparisons of the causes of death (other than the primary cancer) between patients who were neonates at diagnosis and those who were older (>1 month and <2 years).

| Cause of Death | | Age at Diagnosis | | | | | | | | | | | |  |
| --- | --- | --- | --- | --- | --- | --- | --- | --- | --- | --- | --- | --- | --- | --- |
|  |  | Neonatal (<1 month) | | | | | | | Older (>1 month– <2 years) | | | | |  |
|  |  | Count | Col. (%)^*^ | | Survival (mos) | | | |  | |  | Survival (mos) | |  |
|  |  |  |  |  | Mean | | SD | | Count | | Col.  (%)^*^ | Mean | SD |  |
|  | State DC not available or state DC available but no COD | 28 | | 30.8 | | 6 | | 14 | | 86 | 24.6 | 57 | 80 | |
|  | Congenital Anomalies | 25 | | 27.5 | | 3 | | 9 | | 15 | 4.3 | 9 | 14 | |
|  | Certain Conditions Originating during the Perinatal Period^†^ | 21 | | 23.1 | | 0 | | 0 | | 11 | 3.2 | 7 | 12 | |
|  | Other Cause of Death | 6 | | 6.6 | | 14 | | 23 | | 109 | 31.2 | 31 | 61 | |
|  | Diseases of the Heart | 4 | | 4.4 | | 95 | | 175 | | 18 | 5.2 | 102 | 103 | |
|  | Accidents and Adverse Effects | 2 | | 2.2 | | 106 | | 115 | | 9 | 2.6 | 92 | 118 | |
|  | Septicemia | 2 | | 2.2 | | 244 | | 149 | | 11 | 3.2 | 33 | 93 | |
|  | Chronic Liver Disease and Cirrhosis | 1 | | 1.1 | | 0 | | . | |  |  |  |  | |
|  | Bones and Joints | 1 | | 1.1 | | 130 | | . | | 4 | 1.1 | 131 | 41 | |
|  | Brain and Other Nervous System | 1 | | 1.1 | | 202 | | . | | 2 | 0.6 | 49 | 60 | |
|  | Other Infectious and Parasitic Diseases including HIV | . | | . | | . | | . | | 26 | 7.4 | 27 | 51 | |
|  | Pneumonia and Influenza | . | | . | | . | | . | | 22 | 6.3 | 34 | 68 | |
|  | Symptoms, Signs, and Ill-Defined Conditions | . | | . | | . | | . | | 8 | 2.3% | 16 | 19 | |
|  | Cerebrovascular Diseases | . | | . | | . | | . | | 7 | 2.0% | 51 | 72 | |
|  | Acute Myeloid Leukemia | . | | . | | . | | . | | 5 | 1.4% | 28 | 28 | |
|  | Homicide and Legal Intervention | . | | . | | . | | . | | 3 | 0.9 | 146 | 116 | |
|  | Other Diseases of Arteries, Arterioles, Capillaries | . | | . | | . | | . | | 3 | 0.9 | 25 | 20 | |
|  | Other Endocrine Dysfunction, including Thymus | . | | . | | . | | . | | 3 | 0.9 | 37 | 25 | |
|  | Soft Tissue, including Heart | . | | . | | . | | . | | 2 | 0.6 | 245 | 125 | |
|  | Hypertension without Heart Disease | . | | . | | . | | . | | 1 | 0.3 | 25 | . | |
|  | In Situ, Benign, or Unknown Behavior Neoplasm | . | | . | | . | | . | | 1 | 0.3 | 2 | . | |
|  | Kidney and Renal Pelvis | . | | . | | . | | . | | 1 | 0.3 | 7 | . | |
|  | Nephritis, Nephrotic Syndrome, and Nephrosis | . | | . | | . | | . | | 1 | 0.3 | 4 | . | |
|  | Acute Lymphocytic Leukemia |  | |  | |  | |  | | 1 | 0.3 | 166 | . | |
|  | Total | 91 | | 100 | | 19 | | 62 | | 349 | 100 | 44 | 74 | |

^*^Percentages in columns were ordered and colored from highest (red) to lowest (blue).

^†^The perinatal period was defined as the period immediately before, during, or less than a month after birth.

**Abbreviations:** DC, Death Code; COD, cause of death; Col. (%), percentage of column total; SD, standard deviation; mos, months

# Supplementary Table 5.

The relative frequency of neuroblastoma diagnoses and 5-year overall survival of patients with that disease.

| Site of Neuroblastoma | Frequency | RF | 5-year overall survival (95% CI) |
| --- | --- | --- | --- |
| Adrenal gland | 86 | 0.49 | 69.7 (59.7-79.7) |
| Connective tissue | 35 | 0.20 | 85.1 (72.9-97.3) |
| Retroperitoneum | 20 | 0.12 | 90 (76.9-100) |
| Mediastinum | 11 | 0.06 | 88.9 (68.3-100) |
| Other^*^ | 22 | 0.13 | 72.7 (54-91.3) |

^*^Other sites included the kidney, vertebral column, nervous system, thorax, abdomen, and unknown.

# Supplementary Table 6.

Survival plateaus^*^ for each ICCC category in the neonatal group.

| **ICCC category** | **Probability of Overall Survival (SE) after Diagnosis^*^** | | | | | | | | | |
| --- | --- | --- | --- | --- | --- | --- | --- | --- | --- | --- |
|  | **6** | **12** | **18** | **24** | **30** | **36** | **42** | **48** | **54** | **60** |
| Leukemias | 49.4 (5.6) | 45.3 (5.6) | 43.9 (5.6) | **41 (5.6)** | 41 (5.6) | 41 (5.6) | 39.1 (5.7) | 39.1 (5.7) | 39.1 (5.7) | 39.1 (5.7) |
| CNS tumors | 34.7(6) | 27.6 (5.7) | 25.6 (5.6) | **23.6 (5.5)** | 23.6 (5.5) | 21.5 (5.4) | 19.3 (5.3) | 15 (4.9) | 15 (4.9) | 15 (4.9) |
| Neuroblastoma | 80.2 (3.1) | 79.5 (3.1) | 78.2 (3.2) | 77.6 (3.2) | **76.8 (3.3)** | 76.8 (3.3) | 76.8 (3.3) | 76.8 (3.3) | 76.8 (3.3) | 76.8 (3.3) |
| Retinoblastoma | **96.3 (3.6)** | 96.3 (3.6) | 96.3 (3.6) | 96.3 (3.6) | 91.7 (5.7) | 91.7 (5.7) | 91.7 (5.7) | 91.7 (5.7) | 91.7 (5.7) | 91.7 (5.7) |
| Renal tumors | **68.8 (11.6)** | 68.8 (11.6) | 62.5 (12.1) | 62.5 (12.1) | 62.5 (12.1) | 62.5 (12.1) | 62.5 (12.1) | 62.5 (12.1) | 62.5 (12.1) | 62.5 (12.1) |
| Soft-tissue tumors | 70.3 (6.4) | 62.8 (7) | **57.8 (7.3)** | 57.8 (7.3) | 57.8 (7.3) | 55.2 (7.5) | 52.4 (7.6) | 52.4 (7.6) | 52.4 (7.6) | 52.4 (7.6) |
| Germ cell tumors | 75.6 (3.3) | **74.9 (3.4)** | 74.9 (3.4) | 74.1 (3.4) | 73.3 (3.5) | 71.5 (3.6) | 70.5 (3.7) | 69.5 (3.8) | 69.5 (3.8) | 68.3 (3.9) |

^*^The point at which survival probability did not change during the following 6 months was used to determine the start of the survival plateau.
^*^Survival probabilities were calculated every 6 months for each ICCC group until 5-year survival. ICCC groups with fewer than 10 patients were removed to avoid small-sample bias. Time points are noted as months from diagnosis, and survival plateaus for each ICCC category are noted in bold.

# Supplementary Table 7.

Common sites of primary disease identified by the WHO 2008.

| **Sites** | **N** |
| --- | --- |
| Soft tissue, including the heart | 181 |
| Other endocrine, including thymus | 89 |
| Brain | 88 |
| Blood | 85 |
| Miscellaneous | 33 |
| Eye and orbit | 32 |
| Retroperitoneum | 25 |
| Kidney and renal pelvis | 21 |
| Trachea, mediastinum, and other respiratory organs | 17 |
| Bones and joints | 9 |
| Liver | 7 |
| Cranial nerves and other nervous system | 6 |
| Tongue | 3 |
| Melanoma of the skin | 2 |
| Other, nonepithelial skin | 2 |
| Peritoneum, omentum, and mesentery | 2 |
| Urinary Bladder | 2 |
| Gum and other mouth | 1 |
| –Lymph node (Hodgkin) | 1 |
| Lung and bronchus | 1 |
| –Lymph node (Non-Hodgkin) | 1 |
| Oropharynx | 1 |
| Other oral cavity and pharynx | 1 |
| Ovary | 1 |
| Pancreas | 1 |
| Small intestine | 1 |
| Testis | 1 |
| Vulva | 1 |
| Total | 615 |

Abbreviations: WHO, World Health Organization

# Supplementary Figure 1.

Survival by study groups by year of diagnosis. An “event” is defined as death due to cancer OR other causes.

| **Leukemias and Lymphomas** (P = 0.69) |
| --- |
| 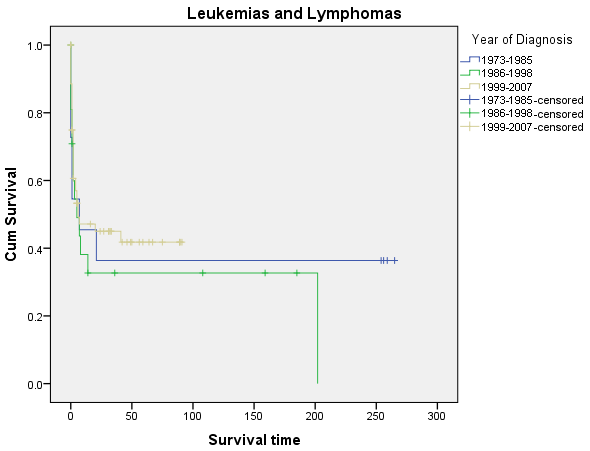 |
| **CNS Tumors** (P <0.005) |
| 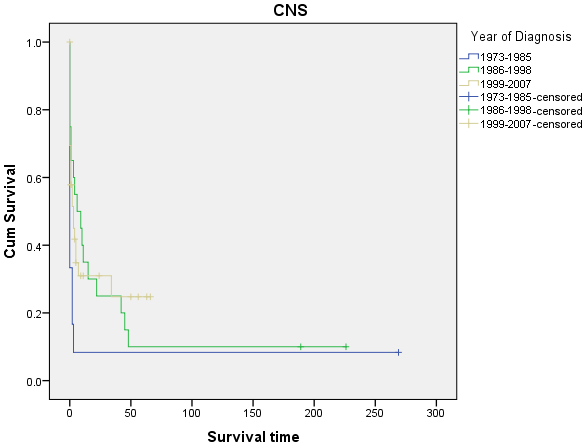 |
| **Solid Tumors** (P >0.5) |
| 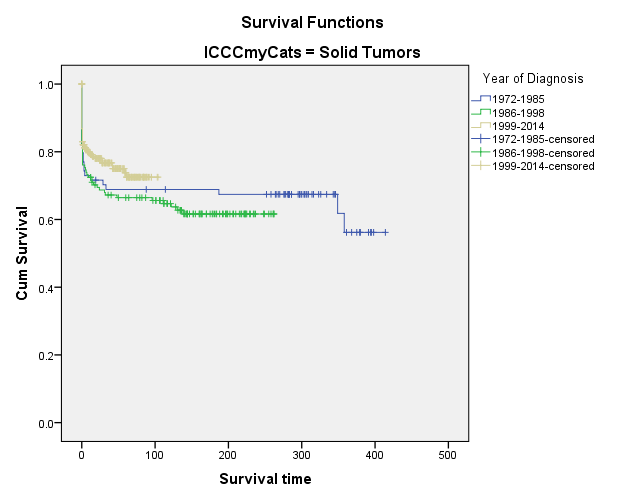 |

# Supplementary Figure 2.

Comparison of cancer- vs. general survival on the basis of age at diagnosis and tumor category.

| **Death from any cause** | **Cancer-related** |
| --- | --- |
| **Leukemias and Lymphomas** | |
| 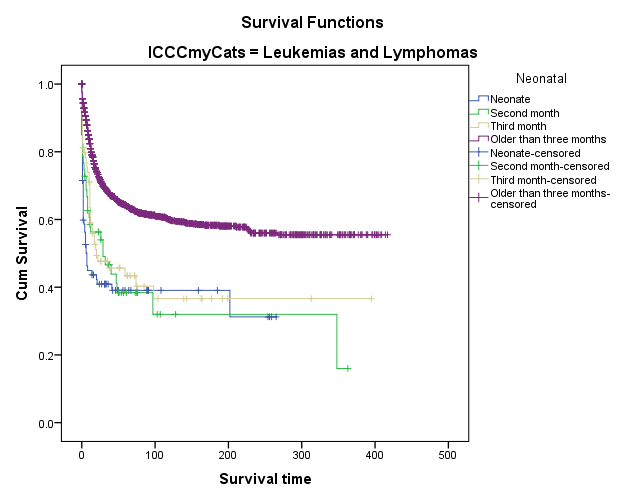 | 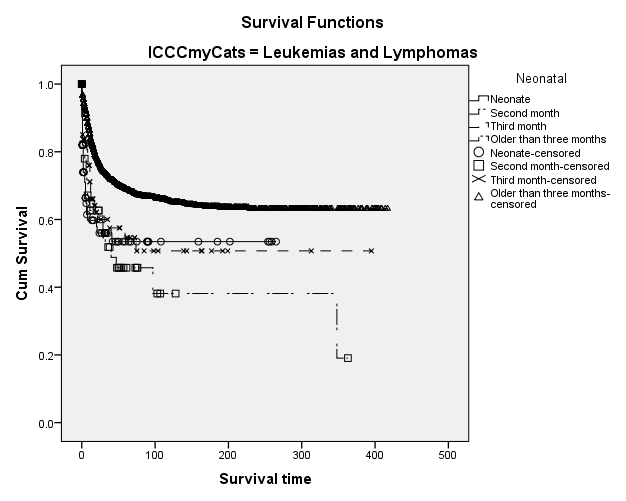 |
| **CNS Tumors** | |
| 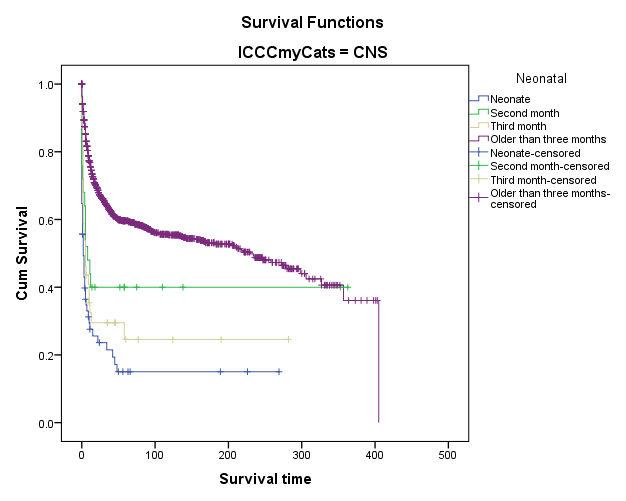 | 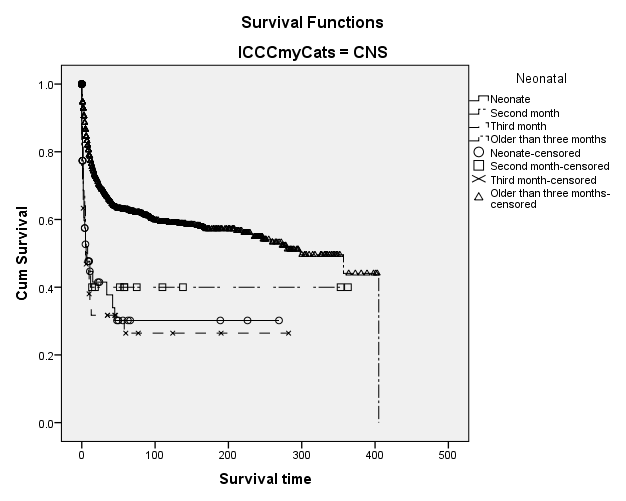 |
| **Solid tumors** | |
| 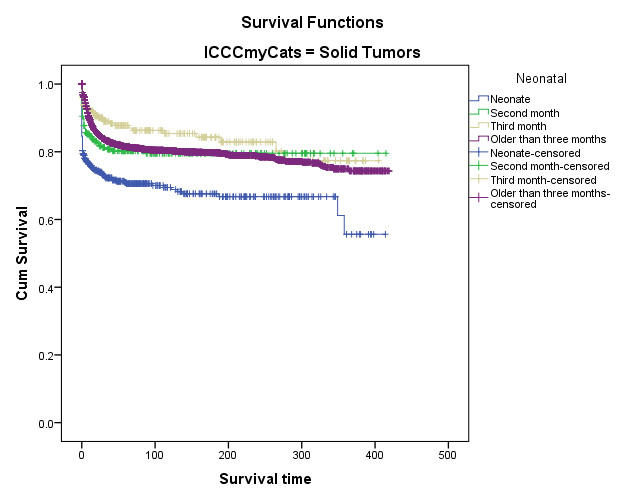 | 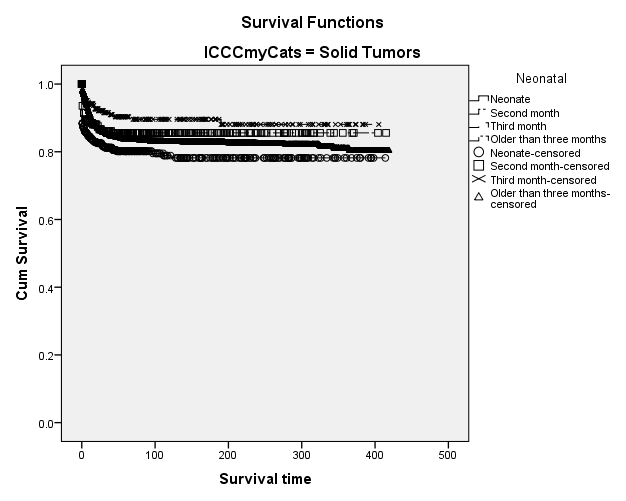 |

# Supplementary Figure 3.

Comparisons of the pattern of intervention between neonatal and older patients over the last 3 decades.

| **Patients older than 1 month** | **Neonatal Patients** |
| --- | --- |
| **Leukemias and Lymphomas** | |
| 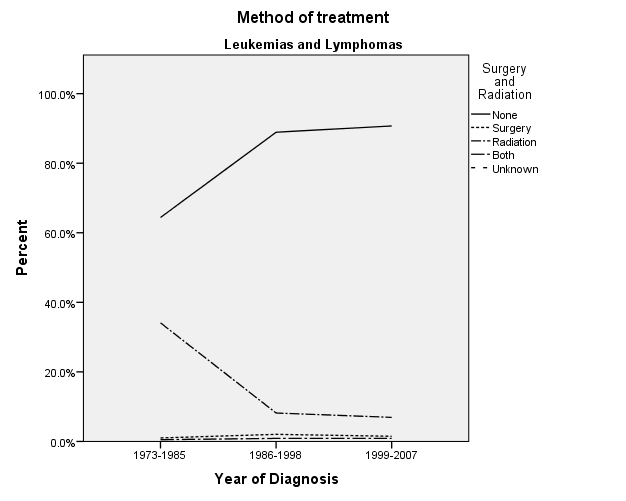 | 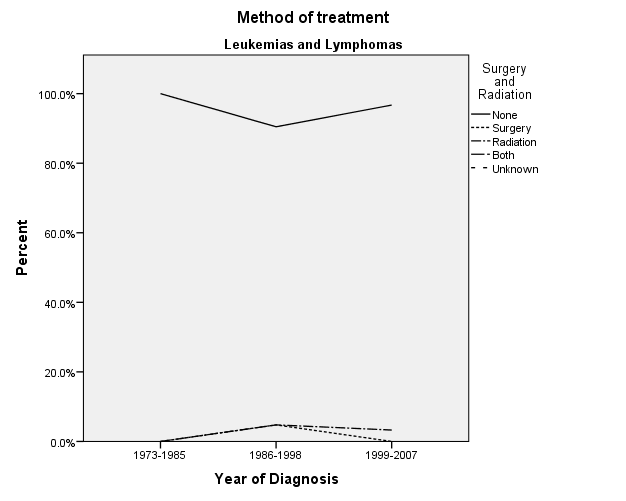 |
| **CNS Tumors** | |
| 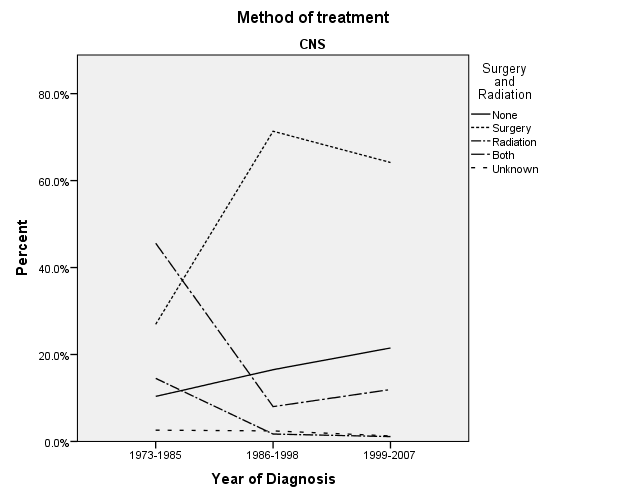 | 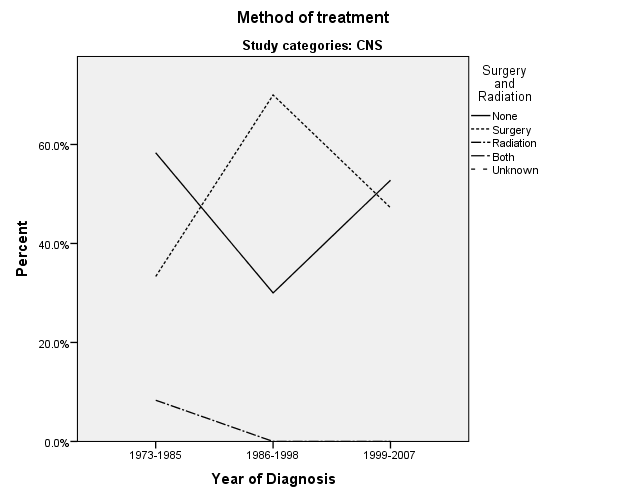 |
| **Solid Tumors** | |
| 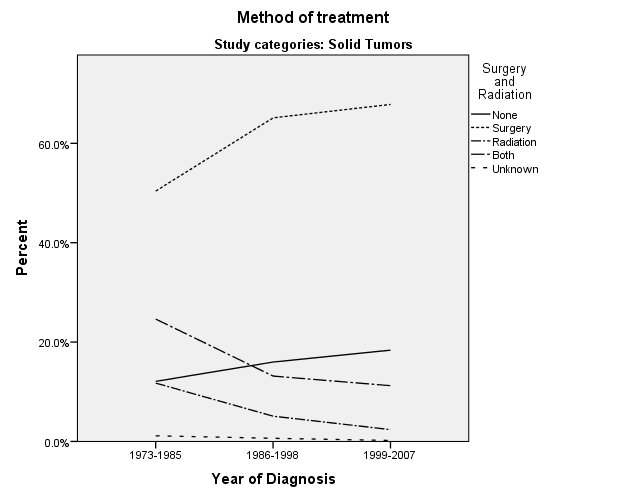 | 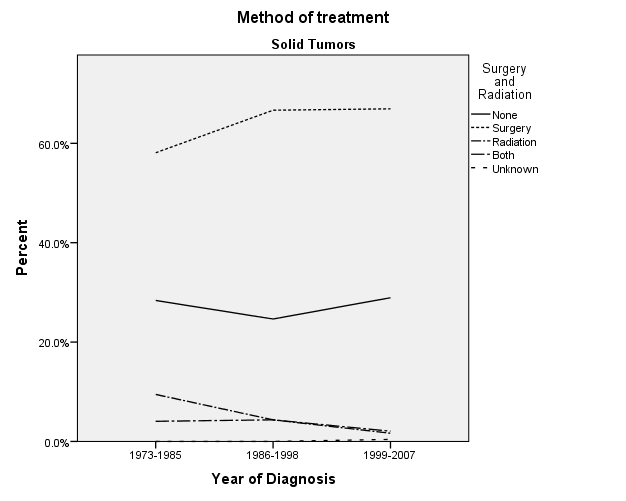 |
